# Multiple Coronary Fistulas as a Rare Cause of Stable Angina Pectoris

**DOI:** 10.1155/2022/9372295

**Published:** 2022-03-04

**Authors:** Jasna Čerkez Habek, Tea Friščić, Jozica Šikić, Marko Perčić, Dean Strinić, Daria Ljubas Perčić

**Affiliations:** ^1^Department of Cardiology, Clinic of Internal Medicine, “Sveti Duh” University Hospital, School of Medicine, University of Zagreb, Zagreb, Croatia; ^2^Department of Neonatology, Clinic of Gynecology and Obstetrition, “Sveti Duh” University Hospital, School of Medicine, University of Zagreb, Zagreb, Croatia

## Abstract

Congenital coronary artery-left ventricular multiple microfistulas (CA-LVMMFs) are rare anomalies in adults. They are more often found in female patients, and they usually originate from the distal segments of the coronary arteries, but they can originate from a proximal segments of a coronary arteries, and these patients are likely to be identified and treated in the pediatric age group. They are mostly asymptomatic. When symptoms and complications occur, they include angina, myocardial infarction, atrial heart failure, arrhythmias, and endocarditis. The management of CA-LVMMFs is controversial, but it is generally agreed that conservative medical management is the primary treatment of choice. Our case describes a rare form of CA-LVMMFs draining into the left ventricle in a female patient presenting with fatigue, atypical anginal symptoms, atrial fibrillation, and premature ventricular complexes, without concomitant obstructive coronary artery disease.

## 1. Introduction

Coronary artery fistulas (CAFs), congenital or acquired due to trauma and iatrogenic causes, are defined as a direct communication of a coronary artery with a cardiac chamber, great vessel, or other vascular structure, bypassing the myocardial capillary bed [1]. Incidence of CAFs is speculative since many are never detected or detected while imaging for another indication. Angiographic series have revealed that the frequency of CAFs in adults is approximately 0.1-0.8% [2, 3]. CAFs have two distinctive angiographic appearances, which could be solitary or multiple and drain into different vascular bed/cardiac chambers [4]. Most CAFs are small, and the patient is usually asymptomatic, but when symptoms and complications occur, they include angina, myocardial infarction, heart failure, arrhythmias, and endocarditis [5]. The management of CAFs is controversial, and recommendations are based on cases or small retrospective series [2]. We report a rare case of CA-LVMMF without concomitant coronary artery disease (CAD).

## 2. Case Presentation

**Figure 1:**
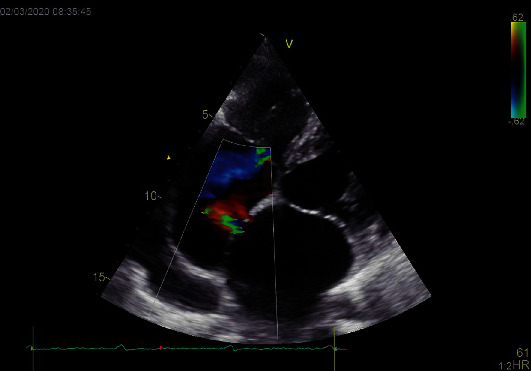
Atrial septal defect with left to right shunt. Both atria are enlarged. Tricuspid regurgitation jet is also visible.

**Figure 2:**
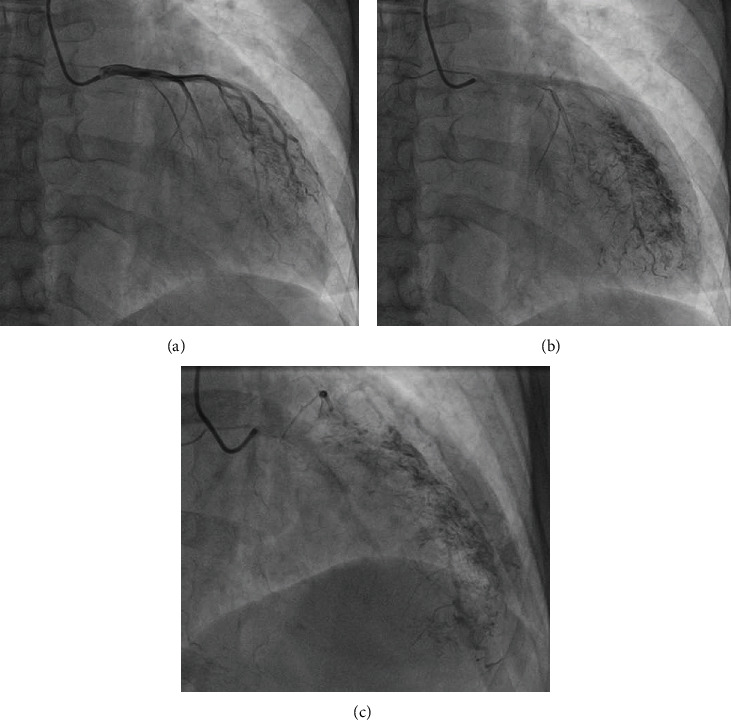
(a) Left coronary artery. (b and c) Multiple microfistulas draining in the left ventricle.

**Figure 3:**
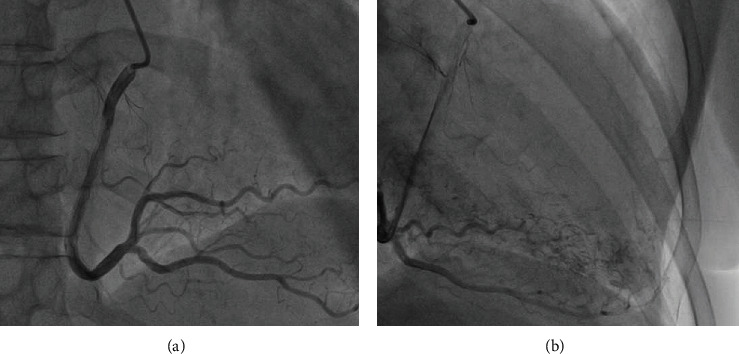
(a) Right coronary artery. (b) Multiple microfistulas draining in the left ventricle.

A 70-year-old female patient with a medical history of chronic myeloid leukemia that was diagnosed five years ago, chronic obstructive pulmonary disease, peripheral artery disease (PAD) with a recently implanted stent in the left superficial femoral artery, permanent atrial fibrillation (AF), and ventricular extrasystole was admitted to the emergency department because of complaints of fatigue, dyspnea and constant, and retrosternal chest pain not related to exertion, described as heaviness which started a week ago. Her chronic therapy already included apixaban and clopidogrel because of PAD and AF. ECG showed AF with single premature ventricular contraction (PVC) and no significant changes in the ST segment. Physical examination was normal. Her blood pressure was 150/100 mmHg, and her heart rate was 85/minute. The laboratory test results were with in normal range, including the troponin level. Coronary angiography has been indicated, and she was admitted to cardiology department. Echocardiography showed normal global and segmental systolic function of the left and right ventricle (Simpson method ejection fraction was 55% and TAPSE 22 mm), moderate diastolic dysfunction of the left ventricle, enlarged left and right atria (LAA 28 cm2 and RAA 30 cm2), mild dilatation of the right ventricle, moderate mitral regurgitation (RV 40 mL and ERO 0.3) due to degenerative alterations of posterior mitral leaflet, and moderate/severe tricuspid regurgitation with RVSP of 50 mmHg. In the area of fossa ovalis, an atrial septal defect (ASD) with left to right shunt was detected with a Qp : Qs 1.3 (Figure 1). Coronary angiography excluded significant obstructive CAD (Figures 2(a) and 3(a)) but showed left ventricle opacification through extensive multiple CA-LVMMF in the late phase of contrast injection to the left coronary arteries (Figures 2(b) and 2(c)) and the right coronary artery (Figure 3(b)). Patient had no chest pain during the hospitalization and was discharged with apixaban, clopidogrel, metoprolol, ramipril, and amlodipine. Two months after the discharge, she had an embolectomy of the right superficial femoral artery, despite apixaban and clopidogrel. On the follow-up in the cardiology clinic, she had no chest pain or palpitation, with occasional dyspnea and 736 PVC in Holter monitor; clopidogrel was excluded from the therapy.

## 3. Discussion

Congenital coronary artery-left ventricular multiple microfistulas (CA-LVMMFs) have infrequent anomalies in adults. CAFs develop early in the embryogenesis, between the sixth and eighth week of gestation, when enlargement of the capillary network from which coronary arteries develop occurs [6]. Approximately 10-30% of patients with CAF also have another congenital cardiovascular anomaly such as tetralogy of Fallot, patent ductus arteriosus, and atrial septal defect [7]. They are more often found in female patients originating from the distal segment of the coronary arteries, but they can originate from a proximal segment of a coronary artery, they are usually larger, and these patients are likely to be identified and treated in the pediatric age group [8]. CA-LVMMFs can lead to angina pectoris and coronary insufficiency even without CAD. Younger patients are generally asymptomatic, but CAF-related symptoms are secondary to fistula size and the volume shunted through, rather than age alone. Typical symptoms are dyspnea, fatigue, palpitation, and angina [9]. According to a Dutch survey of coronary artery fistulas in adult cardiology, the population main-presenting symptoms were angina pectoris and dyspnea in 70% of the patients, ECG showed pathologic changes in 75%, chest X-ray revealed cardiomegaly in 38% of the patients, and congestive heart failure was documented in 10% of the patients. The origin of the fistulas was the left coronary artery in 71% and the right coronary artery in 29% with majority (97%) originating from the mid or distal segments of the coronary vessels [8]. According to Levin et al. 3% of solitary CAFs terminate into the left ventricle [10]. Angina in the absence of CAD is thought to be a consequence of the “steal” phenomenon, which induces ischemia by diverting blood from the high-resistance myocardial capillary bed into the low-resistance fistula [10]. Patients with multiple fistulas in the left ventricle usually present with typical or atypical anginal symptoms [5]. The hemodynamic consequences of CAF vary depending on shunt size, shunt site, and other underlying cardiac diseases. A coronary fistula that drains to the left atrium results in no left to right shunt but causes a volume load similar to mitral regurgitation. Similarly, a coronary artery fistula that drains to the left ventricle produces hemodynamic changes similar to aortic insufficiency [3]. Heart failure is more likely to be seen in adults with concomitant CAD and AF [8]. Surgical strategy for CA-LVMMFs has sporadically been reported in the literature, but patients with multiple fistulae draining into LV without any significant volume overload are generally treated conservatively. Medical management is the primary treatment of choice [8].

## 4. Conclusion

Our case describes a rare form of CA-LVMMFs draining into the left ventricle and ASD in a female patient presenting with fatigue, atypical anginal symptoms, AF, and PVCs, without concomitant CAD or myocardial infarction who has been treated without any specific therapy.

## Data Availability

Readers can access the data supporting the conclusions of the study by direct contact with the corresponding author. All available data the authors considered relevant to the case report were released.
